# Combining nitrate with biochar to reduce methanogenesis in vitro

**DOI:** 10.3168/jdsc.2026-1040

**Published:** 2026-09-07

**Authors:** A. Kolganova, R. Lal, Y. Roman-Garcia, K.E. Mitchell, M. Minnema, J.L. Firkins

**Affiliations:** 1 School of Environment and Natural Resources, CFAES Rattan Lal Center for Carbon Management and Sequestration, The Ohio State University, Columbus, OH 43035; 2 Cargill Animal Nutrition, Innovation Campus, Elk River, MN 55330; 3 Elanco Animal Health Inc., Greenfield, IN 46140; 4 Carbind Labs, Visalia, CA 93274; 5 Department of Animal Sciences, The Ohio State University, Columbus, OH 43035

## Abstract

Enteric fermentation significantly contributes to climate change through the release of CH_4_, which also represents a metabolizable energy loss for the animal. Biochar used in the study is produced from gasification of wood waste and contains redox-active elements; therefore, we hypothesized biochar would inhibit CH_4_ production. In preliminary studies, biochar retained up to 70% of dosed NO_3_^−^ when passed through a filter. Thus, dosed NO_3_^−^ was hypothesized to interact and accentuate biochar's inhibition of CH_4_ production in vitro. A randomized complete block design was used with 200-mL flasks of buffered, anaerobic rumen fluid incubated for 24 h at 39°C in 3 random incubation blocks with 10 duplicated treatment flasks per block that were assessed by orthogonal comparisons using a mixed model. Diets were low- and high-forage both without and with biochar (5.14% of DM) and without or with NO_3_^−^ (3.85% of DM); an additional low-forage diet contained almond hulls (16% of DM) as a forage substitute, without or with biochar. Gas production was measured using gas-impermeable balloons, and CH_4_ was analyzed using a sensor. Main effects of biochar and NO_3_^−^ in the high-forage diet reduced CH_4_ production by 84% and 70%, respectively. The ratio of CH_4_ to NDF degradability decreased for the main effect of biochar administered with the high-forage diet. The combination of biochar and NO_3_^−^ did not surpass any of the individual treatments in lowering CH_4_ concentration. Adding almond hulls did not affect CH_4_ production. Neutral detergent fiber degradability and acetate:propionate ratio were decreased by NO_3_^−^. The main effect of almond hulls increased acetate, butyrate, and propionate but lowered higher-chain molar percentages of volatile fatty acids. Interactions of biochar and NO_3_^−^ for ammonium-N concentration in both diets support the hypothesis of a biochar matrix mediating NO_3_^−^ reduction as an H_2_ sink that is alternate to CH_4_, and minimal residual NO_3_^−^ and NO_2_^−^ suggest complete reduction by 24 h. Microbial N production did not differ among treatments. Although biochar and NO_3_^−^ did not interact for most measurements, further study with longer microbial adaptation is needed to explore potential positive interactions with lower dosages.

## Graphical Abstract

**Figure:**
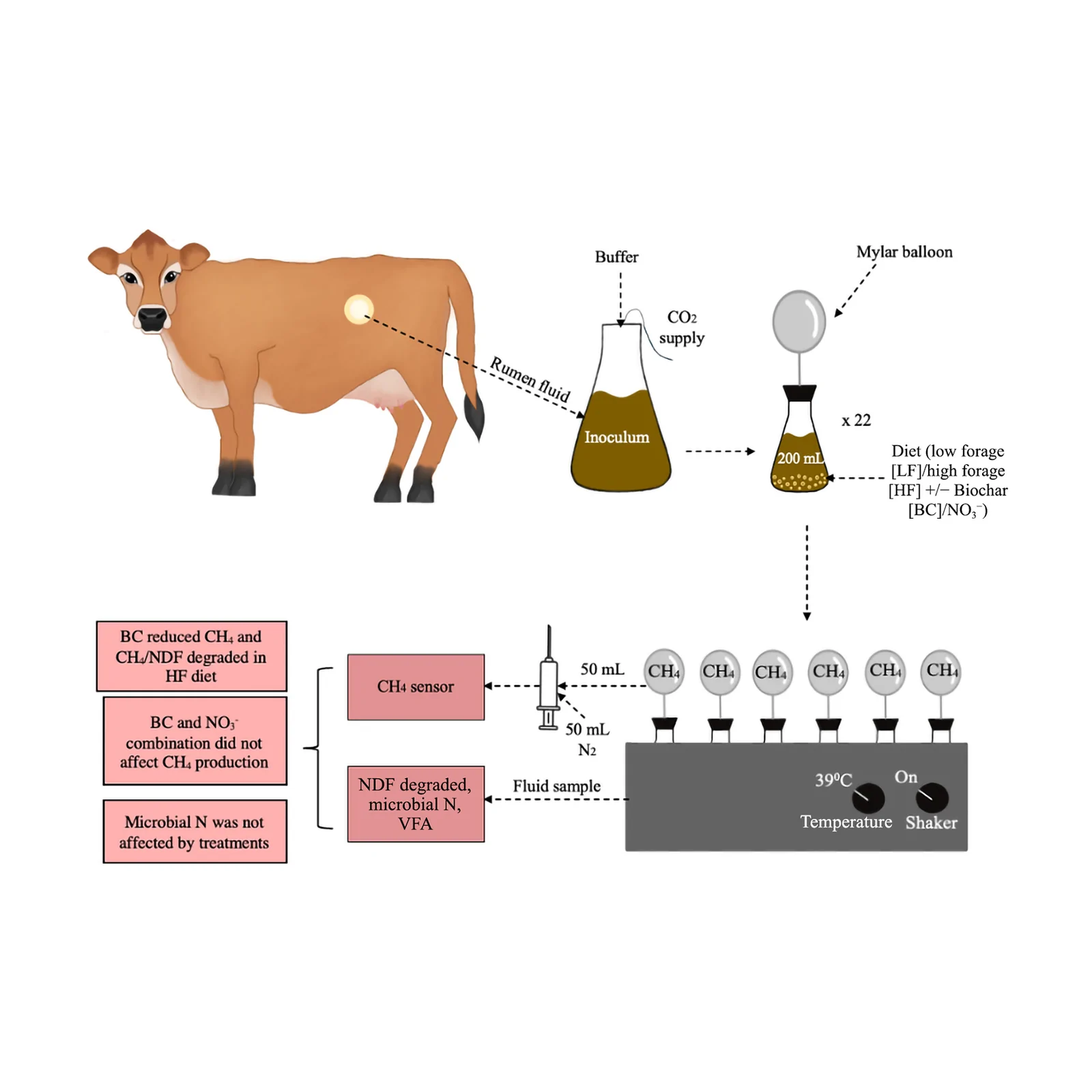

The global warming potential of methane (CH_4_) is estimated to be 28 to 36 times higher than that of CO_2_ (Vallero, 2019). Ruminants, including dairy cattle, are responsible for ∼16% of global CH_4_ emissions (Tseten et al., 2022). Enteric CH_4_ production also represents a loss of dietary metabolizable energy, particularly when H_2_ is emitted during CH_4_ suppression rather than being redirected into more reduced fermentation end products that could otherwise improve feed efficiency. Developing strategies to control and manipulate ruminal fermentation to reduce CH_4_ production in dairy cattle is crucial for improving feed energy utilization and mitigating climate change. In the rumen, aqueous H_2_ serves as the main substrate for methanogenesis, although certain diets increase the flux of methanogenesis from methylamines and other sources (Greening et al., 2019).

Biochar (**BC**) is produced via biomass heating at high temperatures while retaining a high organic matter matrix (Lehmann and Joseph, 2024). The concept of using BC to suppress ruminal methanogenesis is based on its physicochemical characteristics (Honan et al., 2021). Redox-active chemical elements and functional groups on its surface allow BC to mediate electron flow by exchanging electrons from carriers in microbial membranes with surrounding sinks (Mood et al., 2024). High porosity and surface area enable BC to retain chemical ions while providing additional habitat for microbes (Chen et al., 2017; Bolan et al., 2023). However, the effect of BC on CH_4_ production in the rumen is not fully understood because of lack of experiments and a wide variety of BC exhibiting different physicochemical properties.

The BC used in the study is a product of wood waste (e.g., wood chips, sawdust, bark) gasification at above 800°C. The particle size distribution of BC was <0.5 mm, indicating high porosity and consistent with a high surface area (423 m^2^/g, on a DM basis), estimated by Control Laboratories (Watsonville, CA) using American Society for Testing and Materials (**ASTM**) method D1762-84. The product had low ash content (5.8% of total DM) and high content of organic C (83.7% of total dry mass), as determined by Control Laboratories using ASTM D-1762-84 and dry combust-ASTM D 4373 methods respectively (ASTM International, 2007, 2021).

The chemical composition analysis of BC was performed using EPA methods 3050B and EPA6020B (US EPA, 1996, 2014). The BC contained 3.6% CaCO_3_. Our BC was primarily composed of redox-active elements (Na, 56%; Fe, 31%; Mn, 6%; and Cl, 4%; DM basis). These elements contribute to redox-active functional groups on the BC surface. In particular, Fe-containing functional groups served as electron acceptors or donors, depending on the oxidation state of Fe, for the gut bacterium *Enterococcus faecalis* (Keogh et al., 2018).

Supplementing nitrate (NO_3_^−^) inhibits ruminal methanogenesis; however, because of a decreased DMI and possible accumulation of NO_2_^−^, the adoption of this strategy has been limited (Lee and Beauchemin, 2014). We conducted a preliminary study to test the ability of BC to retain NO_3_^−^ when added to a NaNO_3_ solution following the procedure described by Jatana et al. (2020) with minor adjustments. The BC retained 70% of NO_3_^−^ (data not shown). The retention of NO_3_^−^ by BC increases its proximity to BC particles. The ability of BC to act as an electron shuttle or provide chemical bridging through redox-active surface groups may facilitate NO_3_^−^ reduction and contribute to NO_2_^−^ retention (Sanford et al., 2019). Therefore, we hypothesized that NO_3_^−^ would be embedded in the BC matrix and nonadditively interact with BC to suppress CH_4_ production.

The study was conducted as a randomized complete block design with 3 inoculation blocks using 10 treatments duplicated in individual 200-mL flasks containing buffered, anaerobic rumen fluid. Each flask received 3.89 g of dietary DM. Experimental diets consisted of a low-forage (**LF**) and a high-forage (**HF**) diet based on orchardgrass and concentrate mixtures, along with an LF diet supplemented with the highly degradable fiber source almond hulls (**AH**; 16% of DM) replacing corn to approximate the fiber in HF. The forage-to-concentrate ratios were 40:60 for the LF diets and 60:40 for the HF diet. Crude protein was maintained at 15.4% of DM across all diets. The NDF of LF, HF, and AH diets was 33.6%, 44.3%, and 31.5% respectively (data not shown). The LF and HF diets each included 4 treatments in a 2 × 2 factorial arrangement, with or without BC (5.14% of DM) and with or without NO_3_^−^ (3.85% of DM). The LF diet containing AH included 2 treatments, with or without BC, and did not include NO_3_^−^ supplementation. Treatment inclusion rates were selected to maximize the likelihood of detecting the main effects of BC and its potential interaction with NO_3_^−^, providing proof-of-concept evidence while minimizing the risk of Type II error. The buffer solution was prepared according to Goering and Van Soest (1970) and continuously sparged with O_2_-free CO_2_ for 24 h before inoculation.

Rumen fluid was obtained from 2 cannulated lactating Jersey cows following an approved IACUC protocol. The cows were fed a standard lactation diet including 39% corn silage, 13.3% alfalfa hay, 20.0% ground corn, 9.9% wet brewers grains, 7.7% soybean meal, 4.6% AminoPlus (soybean meal processed to be higher in RUP; AGP Ag Processing Inc.), 0.9% Megalac (calcium salt of fatty acids; Church and Dwight), 0.9% molasses, and the remaining 3.4% containing vitamins and minerals (data not shown).

Collected rumen fluid was squeezed through 4 layers of cheesecloth into warmed, insulated 500-mL bottles, and transported to the laboratory at 39°C within 30 min. In the laboratory, ruminal fluid was mixed and blended for 30 s with a Waring blender, bubbled with O_2_-free CO_2_, and diluted 1:4 with buffer before anaerobically inoculating the 200-mL flasks. After flushing the headspace with CO_2_, gas-impermeable balloons were attached on top of each flask to maintain anaerobiosis while releasing gas to allow buffering. The flasks were placed into a 39°C water bath with a shaking mechanism for 24 h.

At the end of the 24-h incubation, gas production was measured by volume of the sealed gas-impermeable balloons manually using the water displacement method. The CH_4_ concentration was measured by injecting a 100-mL gas syringe with 50 mL of gas sample diluted with 50 mL of N_2_ into a CH_4_ sensor (Micro-Oxymax Respirometer, Columbus Instruments, Columbus OH). A 4-mL aliquot of fluid was used for VFA analysis by GC (Roman-Garcia et al., 2021). A 25-mL aliquot was taken for NO_3_^−^ and NO_2_^−^ analyses via the flow injection method (Giné et al., 1980). The NDF of feed and residue was determined following the procedure described by Van Soest et al. (1991) using Na_2_SO_3_ and heat-stable amylase. Microbial N production was evaluated by dosing 10% enriched ^15^(NH_4_)_2_SO_4_ and ^15^NaNO_3_ and further elemental analysis of residue and bacteria harvested and analyzed as described by Wenner et al. (2020).

A mixed model included incubation period as a random effect in R Studio (version 2024.04.2-764, Posit Software, 2024). Nine orthogonal contrasts were specified a priori to test: the effect of BC within each level of NO_3_^−^ and forage (LF vs. HF), the effect of NO_3_^−^ within each level of BC and forage, the BC × NO_3_^−^ interaction within each forage level, and the effect of AH and its interaction with BC are shown in footnotes in Table 1. *P* ≤ 0.05 was considered significant and 0.05 < *P* ≤ 0.10 was considered a tendency for significance.

**Table 1:** Means of the 24-h batch culture incubations with low-forage (LF) and high-forage (HF) diets with or without biochar, nitrate, and almond hulls

Item	Treatment^1^										SEM
	LF						HF				
	CON	BC	NO_3_^−^	BC + NO_3_^−^	AH	AH + BC	CON	BC	NO_3_^−^	BC + NO_3_^−^	
CH_4_,^2^, ^3^ μmol	2.97	0.65	0.78	0.24	1.05	0.68	5.80	0.76	1.55	0.41	0.1
NDFD,^3^ %	33.3	28.7	27.4	29.7	30.5	27.6	37.0	37.5	34.6	33.3	3.9
CH_4_/NDFD,^2^, ^3^, ^4^ μmol/g	8.97	2.12	2.83	0.77	3.58	2.65	15.68	2.37	4.43	1.36	0.30
Total VFA,^5^ mmol/L	122	110	109	120	108	111	110	95	104	91	9
VFA, mol/100 mol											
Acetate^5^, ^6^, ^7^	62.1	61.2	58.6	60.0	63.5	63.8	63.2	62.8	65.6	62.3	1.9
Propionate^5^, ^6^, ^7^	19.0	19.9	24.7	23.9	19.7	21.1	20.5	20.6	21.1	24.8	1.6
Butyrate^5^, ^6^, ^7^	15.6	15.7	13.9	13.5	13.8	12.4	13.4	13.6	11.2	10.7	0.8
Caproate^4^, ^5^, ^7^	1.25	1.16	0.87	0.8	1.06	0.90	1.10	1.13	0.82	0.59	0.15
Valerate^5^	2.02	1.97	1.95	1.82	1.96	1.80	1.83	1.93	1.78	1.64	0.11
Isobutyrate^5^, ^6^	1.10	0.94	0.76	0.91	0.83	0.72	0.76	0.73	0.78	0.56	0.15
2-Methylbutyrate^5^, ^6^	1.14	0.98	0.94	1.10	0.86	0.74	0.78	0.78	0.94	0.35	0.26
Isovalerate^5^, ^6^, ^7^	1.11	0.94	0.67	0.83	0.82	0.68	0.69	0.67	0.72	0.40	0.16
NH_4_-N,^2^, ^3^, ^4^, ^5^, ^6^, ^7^, ^8^, ^9^, ^10^ mg/dL	43.9	47.0	29.3	20.5	42.6	31.6	41.3	38.0	47.2	26.2	1.5
Microbial N, mg	39.1	30.2	34.0	39.7	44.7	43.5	30.3	30.5	39.5	40.3	10.4
Microbial N/NDFD, mg/g	49.7	26.2	39.6	34.1	49.5	34.9	31.5	24.4	39.6	33.5	10.2
Microbial N from NH_3_,^7^, ^10^ %	71.9	53.8	35.6	35.5	45.6	63.7	55.9	37.7	35.8	35.9	9.7
Microbial N from NH_3_,^10^ mg	29.4	15.5	12.3	14.2	21.0	30.2	16.6	12.5	14.0	15.0	7.4
pH^4^, ^5^, ^6^, ^7^, ^9^	6.24	6.27	6.30	6.28	6.36	6.39	6.38	6.35	6.35	6.35	0.02

1 Treatments: CON = control (no supplement); BC = biochar (5.14% of DM); NO_3_^−^ = nitrate (3.85% of DM); BC + NO_3_^−^ = biochar and nitrate combination (5.14% and 3.85% of DM, respectively); AH = almond hulls (16%) in LF (40%) and HF (60%) diets; AH + BC = almond hulls and biochar combination (16% and 5.14% of DM, respectively). NDFD = NDF degraded.

2 Main effect of BC in HF treatments (CON and NO_3_^−^ vs. BC and BC + NO_3_^−^ in HF diet, *P* ≤ 0.03).

3 Main effect of NO_3_^−^ in HF treatments (CON and BC vs. NO_3_^−^ and BC + NO_3_^−^ in HF diet, *P* ≤ 0.10).

4 Interaction of BC × NO_3_^−^ in HF treatments (CON and BC + NO_3_^−^ vs. BC and NO_3_^−^ in HF diet, *P* ≤ 0.10).

5 Main effect of AH in LF treatments (CON and BC vs. AH and AH + BC in LF diet, *P* ≤ 0.06).

6 Contrast between LF and HF (CON, BC, NO_3_^−^, and BC + NO_3_^−^ in LF diet vs. CON, BC, NO_3_^−^, and BC + NO_3_^−^ in HF diet, *P* ≤ 0.09).

7 Main effect of NO_3_^−^ in LF treatments (CON and BC vs. NO_3_^−^ and BC + NO_3_^−^ in LF diet, *P* ≤ 0.04).

8 Main effect of BC in LF treatments (CON and NO_3_^−^ vs. BC and BC + NO_3_^−^ in LF diet, *P* = 0.07).

9 Interaction of BC × NO_3_^−^ in LF treatments (CON and BC + NO_3_^−^ vs. BC and NO_3_^−^ in LF diet, *P* = 0.01).

10 Interaction of AH × BC in LF treatments (CON and AH + BC vs. BC and AH in LF diet, *P* ≤ 0.08).

Adding AH to the LF diet did not affect CH_4_ production (Table 1). Main effects of BC and NO_3_^−^ in the HF diet decreased (*P* ≤ 0.04) CH_4_ production by 87% and 73%, respectively. In the HF diet, BC did not affect NDF degradability (**NDFD**), but NO_3_^−^ decreased (*P* = 0.04) NDFD by 5.9%. The CH_4_/NDFD was decreased (*P* = 0.03) by the main effect of BC by 85% in HF diet. Although CH_4_ was not affected by treatments in LF diets, NO_3_^−^ decreased (*P* = 0.04) acetate but increased (*P* = 0.01) propionate molar percentage. The main effect of AH tended to increase (*P* ≤ 0.06) acetate and propionate while lowering butyrate, caproate, valerate, isobutyrate, 2-methylbutyrate, and isovalerate molar percentages. Interactions of BC and NO_3_^−^ reflected decreasing (*P* ≤ 0.02) NH_4_^+^-N concentration, with the greatest reduction occurring when NO_3_^−^ was combined with BC in both HF and LF diets, supporting our hypothesis of a BC matrix facilitating NO_3_^−^ reduction relative to NH_4_^+^ assimilation into microbial N. Minimal residual NO_3_^−^ and NO_2_^−^ concentrations showed complete reduction by the end of our 24-h incubation (0.97 ± 0.09 and 0.046 ± 0.007 mg/L of NO_3_^−^ and NO_2_^−^, respectively, for treatments with added NO_3_^−^; data not shown). Microbial N production was not affected by any treatment. Nevertheless, the main effect of NO_3_^−^ lowered (*P* < 0.01) the percentage of microbial N derived from NH_3_, although this effect lost significance when expressed as absolute microbial N derived from NH_3_. The interaction of AH and BC increased (*P* = 0.02) microbial N derived from NH_3_ on both bases.

Biochar can inhibit CH_4_ production in rumen fluid (Table 1). The mode of action of BC in the rumen is currently a knowledge gap and probably depends on the preparation method; nevertheless, BC might suppress methanogenesis by serving as an extracellular electron shuttle . Redox-active functional groups on BC such as Fe could mediate direct interspecies electron transfer between syntrophic partners (Zhao et al., 2024). Biochar likely can form tight connections between membranes of syntrophic partners to facilitate this electron transfer (Zhang et al., 2025). However, to our knowledge, such systems have not been investigated with mixed ruminal microbes. Because H_2_ is poorly soluble and rapidly diffuses in aqueous environments, efficient interspecies H_2_ in the rumen transfer requires close spatial proximity between H_2_-producing and H_2_-consuming microorganisms (Mackie et al., 2024). Methanogens commonly form close physical associations with H_2_-producing syntrophic microorganisms. Through Ca^2+^- and Fe^3+^-mediated bridging of NO_3_^−^ to negatively charged bacterial cell walls, BC can retain NO_3_^−^ and increase the proximity of NO_3_^−^ to microbial communities occupying its porous structure (Luo et al., 2025). By promoting interspecies electron transfer rather than relying on H_2_ as an electron carrier, BC might redirect reducing equivalents toward alternative electron sinks, such as NO_3_^−^, thereby decreasing H_2_ availability for methanogens.

In the rumen, NO_3_^−^ is an alternate electron sink compared with methanogenesis (Feng et al., 2020). Using the mixed model of Feng et al. (2020) with a slope of −0.911% decrease in CH_4_ adjusted for our 3.85% dose, their model predicts 35% decrease in CH_4_. Our much higher results probably reflect lack of adaptation of microbes to NO_3_^−^. For example, Wenner et al. (2020) reported a 31% decrease in methanogenesis with 1.5% NO_3_^−^, with continuous culture preventing any absorption of NO_3_^−^ and NO_2_^−^ as in our closed system compared with in vivo. Our comparable response for BC to suppress methanogenesis comparably with NO_3_^−^ is also beyond expectations for the role of BC in methanogenesis (Honan et al., 2021). Part of the lower response in the previous authors' meta-analysis is a result of the large variability among types of BC, and our dose was likely higher than doses in prior studies. Thus, we hypothesize that BC can facilitate NO_3_^−^ reduction more completely with much less NO_3_^−^ and especially NO_2_^−^ absorption from the rumen. The combination of BC and NO_3_^−^ did not improve CH_4_ suppression beyond that achieved by either treatment alone and also decreased NH_4_^+^-N availability. However, our current responses lead us to suggest that positive interactions could be detected with lower dosages and increased time for microbial adaptation. Thus, a future study is planned to test the direct effect of both BC and its combination with NO_3_^−^ on CH_4_ production in rumen fluid.

In conclusion, the findings of this batch culture study provide proof-of-concept support for BC to interact with NO_3_^−^ to more completely reduce it to NH_4_^+^ while keeping NO_2_^−^ embedded in its matrix to decrease its absorption or passage from the rumen. Importantly, these high-dose treatments did not impair NDFD or microbial N production. The BC tested here likely possesses a unique set of physicochemical properties that need further assessment to optimize the various BC products available for potential to suppress ruminal methanogenesis. The combination of BC and NO_3_^−^ may provide an effective strategy for suppressing ruminal methanogenesis while allowing lower NO_3_^−^ inclusion rates. This effect may be mediated through enhanced electron exchange among BC, microbial communities, and N-containing redox species, thus facilitating more efficient NO_3_^−^ reduction, limiting H_2_ accumulation and gaseous losses, and directing reducing equivalents toward microbial end products rather than methanogenesis.
